# Management of Coronary Artery Bypass Grafting Using an Arteriovenous Fistula: An Intraoperative Change in the Preoperative Plan

**DOI:** 10.7759/cureus.35517

**Published:** 2023-02-27

**Authors:** Jien Saito, Shinji Kamiya, Yukihide Numata, Hideki Sasaki, Miki Asano

**Affiliations:** 1 Cardiovascular Surgery, Nagoya City University East Medical Center, Nagoya, JPN

**Keywords:** coronary artery bypass grafting (cabg), arteriovenous fistula, intraoperative finding, hd (hemodialysis), internal thoracic artery harvesting site

## Abstract

Regarding coronary artery bypass grafting (CABG) in patients on hemodialysis, in situ internal thoracic artery (ITA) grafting of the left anterior descending artery (LAD) improves survival and freedom from cardiac events. Although a problem with the ITA can possibly occur, using the ITA ipsilateral to an arteriovenous fistula (AVF) in the upper extremity of patients on hemodialysis can cause coronary subclavian steal syndrome (CSSS). CSSS is a condition of myocardial ischemia caused by the diversion of blood flow from the ITA following coronary artery bypass surgery. CSSS has been reported to occur in cases of subclavian artery stenosis, AVF, and low cardiac function.

A 78-year-old man with end-stage renal disease experienced angina pectoris during hemodialysis. The patient was scheduled for CABG, including anastomosis of the left internal thoracic artery (LITA) and LAD. After completion of all anastomoses, the LAD graft demonstrated retrograde blood flow, suggestive of ITA anomalies or CSSS. The LITA graft was transected at the proximal part and anastomosed to the saphenous vein graft with sufficient flow to the high lateral branch eventually.

## Introduction

In situ bilateral internal thoracic artery (ITA) grafts are first-line conduits, even in coronary artery bypass grafting (CABG) in patients on hemodialysis, particularly in those with multivessel disease and aortic calcification [1,2]. However, there is controversy on the use of the ipsilateral ITA with an arm arteriovenous fistula (AVF), regarding coronary subclavian steal syndrome (CSSS). This syndrome causes myocardial ischemia by retrograde blood flow to the in situ ITA graft, even without structural stenosis of the subclavian artery [3]. Moreover, because intraoperative spasms, dissections, or hematomas of the ITA may result in poor flow, estimating CSSS occurrence intraoperatively or predicting it preoperatively can be challenging [4]. Based on our current findings, the feasibility of ipsilateral ITA grafts in patients on hemodialysis with AVF is questionable because there are many intraoperative confounding factors affecting the decision.

## Case presentation

Here, we present the case of a 78-year-old man with end-stage renal disease who experienced angina pectoris during hemodialysis. The patient was started on hemodialysis due to immunoglobulin A nephropathy; no diabetes mellitus was noted. He was referred to our hospital for the management of angina pectoris.

**Figure 1:**
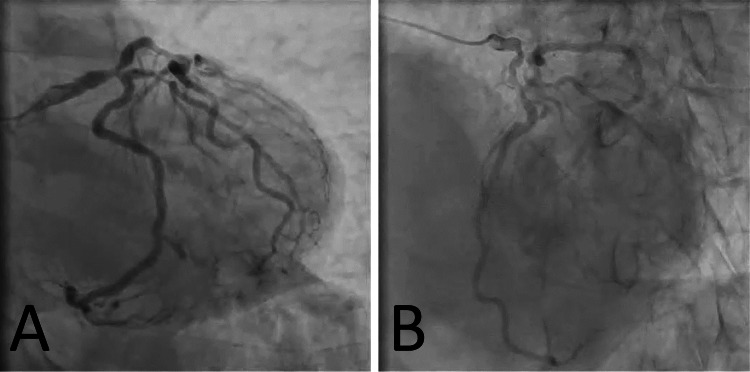
Coronary angiography showing stenosis in different segments of the coronary arteries, according to the American Heart Association Classification of Coronary Artery Segments. Strong stenosis at the LMT to HL/LAD/LCX bifurcation. (A) CAG image showing 75% stenosis in segment #5, 90% stenosis in segment #6, and 90% stenosis in segment #11. The image was taken using RAO 0°, CAU 40° view. (B) CAG image showing 90% stenosis in HL, and 90% stenosis in segment #11. The image was taken using LAO 50°, CRA 25° view. CAG: coronary angiography; LMT: left main trunk; HL: high lateral branch; LAD: left anterior descending artery; LCX: left circumflex artery; RAO: right anterior oblique; CAU: caudal; LAO: left anterior oblique; CRA: cranial

A subsequent coronary angiography showed a two-vessel lesion, including the left main coronary trunk. The degree of stenosis was 75% in segment #5, 90% in segment #6 (instantaneous wave-free ratio [iFR], 0.61; fractional flow reserve [FFR], 0.62), 90% in the high lateral branch (HL), and 90% in segment #11 (iFR, 0.86; FFR, 0.68) (Figure 1). Contrast-enhanced computed tomography (CT) showed no stenosis in both the bilateral ITA and subclavian arteries. In addition, the vessel diameters of the bilateral vertebral arteries were similar. Magnetic resonance angiography (MRA) of the neck showed poor imaging of the left vertebral artery. CT and MRA findings together indicated possible hypoperfusion in the same vessel.

Ultrasonography revealed a relatively high flow volume of the AVF (1,132 mL/minute). The ankle-brachial index (ABI) was 1.20 (right) and 1.04 (left); 169/128 mmHg (% mean arterial pressure [MAP], 52%) for the right upper extremity, 161/120 mmHg (% MAP, 48%) for the left upper extremity, 202/82 mmHg (% MAP, 42%) for the right lower extremity, and 175/132 mmHg (% MAP, 43%) for the left lower extremity. These values were within the standard range with no differences between the upper extremities, indicating no blood flow captured by the AVF.

**Figure 2:**
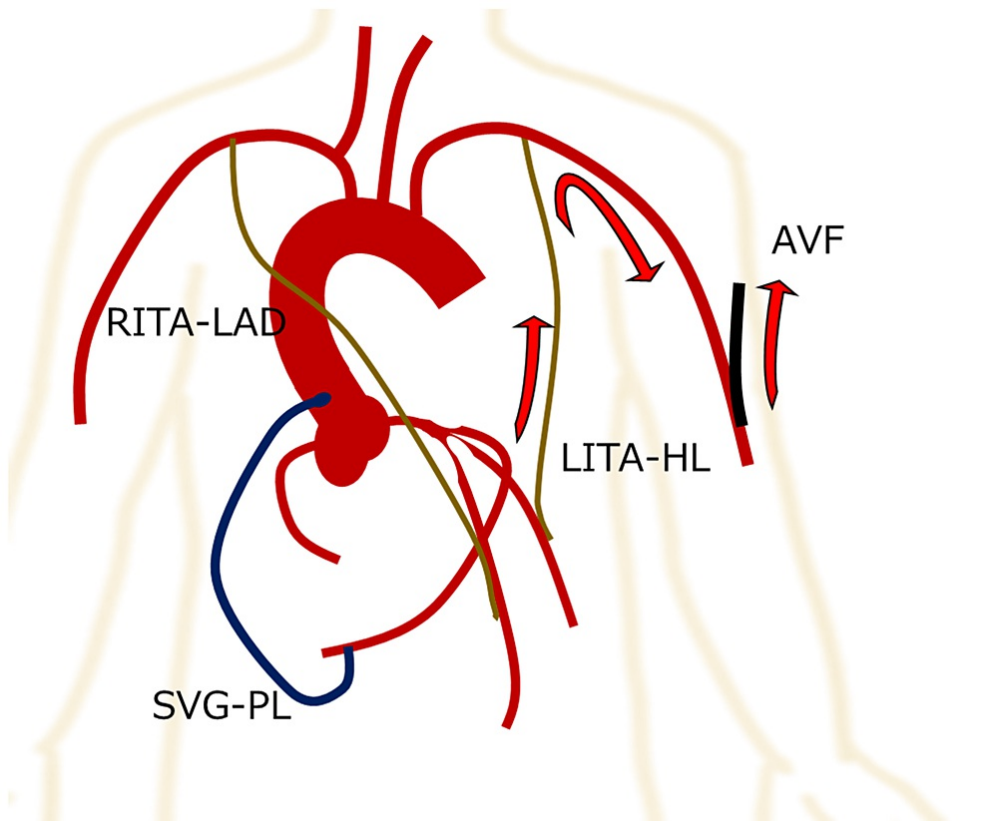
Schematic of surgical findings. Retrograde blood flow from graft to AVF is shown. Image credits: Jien Saito. RITA: right internal thoracic artery; LAD: left anterior descending artery; SVG: saphenous vein graft; AVF: arteriovenous fistula; LITA: left internal thoracic artery; HL: high lateral branch

**Figure 3:**
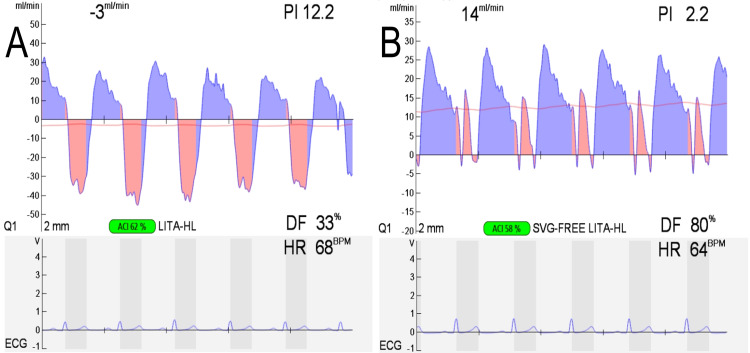
Transit time flowmeter. Reanastomosis of the proximal side of the LITA graft has resulted in a better blood flow pattern. (A) LITA-HL graft before reanastomosis. The blood flow showed a retrograde pattern. (B) Free LITA-LAD graft after reanastomosis. The blood flow rate shows 14 mL/minute and improves to a progressive pattern. LAD: left anterior descending artery; LITA: left internal thoracic artery; HL: high lateral branch

Off-pump CABG, comprising anastomosis of the left internal thoracic artery (LITA) to the left anterior descending artery (LAD), right internal thoracic artery (RITA) to HL, and a saphenous vein graft to the posterolateral branch had been scheduled. Based on the finding that the free flow of in situ LITA was 20 mL/minute but a weak pulsation, and its potential insufficiency for revascularization of LAD with high flow demand, we revised the preoperative plan to anastomose RITA to LAD instead [5]. We also evaluated LITA after anastomosis to HL. After completion of all anastomoses, as feared, the LITA graft demonstrated retrograde blood flow on a transit time flowmeter (TTFM), which suggested the occurrence of ITA anomalies or CSSS (Figure 2, Figure 3, Panel A).

Electrocardiography indicated no changes, including in leads ST-T. Although the TTFM pattern was poor and some intervention was needed, anastomotic stenosis was considered unlikely. If the ITA itself is anomalous, such as with spasms, dissections, or hematomas, the graft may be occluded or improve over time. However, in cases of CSSS, there is a risk of myocardial ischemia, which might further worsen the patient’s condition. Therefore, in our case, the LITA graft was transected at the proximal part and anastomosed to the saphenous vein graft with sufficient flow to reach the HL (14 mL/minute) as a Y-composite graft (Figure 3, Panel B). Postoperative angiography showed good patency of all bypass grafts. The patient was discharged from the hospital 16 days after surgery.

Ethics approval and consent for this case report were waived, as this report was conducted by anonymizing medical institution and patient personal information so that the patient could not be identified. However, written informed consent was obtained from the patient for the publication of this report and accompanying images.

## Discussion

In situ, both ITAs grafted to the LAD improve survival and freedom from cardiac events for CABG in patients on hemodialysis [1,2]. However, ipsilateral AVF may compromise the mortality benefit originally provided by ITA grafting. Although no prognostic value has been demonstrated regarding ipsilateral ITA grafting, the risk of major adverse cardiac events increases due to the effects of AVF during hemodialysis. When using the ipsilateral ITA, the possibility of CSSS should be considered beforehand [6,7].

In this case, the relatively lower free flow of the LITA and higher flow of the AVF indicated that the original blood flow of the LITA might have been drawn into the AVF, even with no laterality of the preoperative ABI [8]. The free flow was estimated to be approximately 20 mL/minute by quadrupling the blood stored for 15 seconds at the transection. Although the free flow of less than 40 mL/minute is considered inadequate, no clear evidence is available [5]. We did not attempt the occlusion test of the AVF during the surgery. Although the remarkable increase in graft free flow by the test might predict CSSS occurrence, it is difficult to affirm that this pathophysiologic state is caused only by these clinical factors. This is partially because the ITA itself may have been abnormal due to intraoperative manipulation. In this condition, it depends on the blood flow balance between a graft and the target coronary arteries [9].

It is not clear whether CSSS is established only by measuring graft blood flow after the completion of a bypass anastomosis. Although predicting CSSS preoperatively or intraoperatively is challenging, it can be caused by excessive AVF or subclavian artery stenosis [7]. Therefore, in patients on hemodialysis with AVF, contralateral ITA is highly recommended to prevent CSSS. Ipsilateral ITA should be used as a free graft, considering the advantages of arterial graft in CABG. Further studies are needed to evaluate the utilization of ITA in concomitant ipsilateral constructed AVF.

## Conclusions

For CABG in a patient on hemodialysis with an arm AVF, the blood flow of AVF and free flow of the ipsilateral ITA are regarded as predictive factors of CSSS occurrence. However, they do not represent decision factors. The ipsilateral ITA may be used as a free graft and not as an in situ graft in cases where there are concerns about ipsilateral ITA blood flow to prevent CSSS occurrence.
